# Preparation and Characterization of a Gastric Floating Dosage Form of Capecitabine

**DOI:** 10.1155/2013/495319

**Published:** 2013-10-29

**Authors:** Ehsan Taghizadeh Davoudi, Mohamed Ibrahim Noordin, Ali Kadivar, Behnam Kamalidehghan, Abdoreza Soleimani Farjam, Hamid Akbari Javar

**Affiliations:** ^1^Department of Pharmacy, Faculty of Medicine, Universiti of Malaya, 50603 Kuala Lumpur, Malaysia; ^2^Institute of Tropical Agriculture, Universiti Putra Malaysia (UPM), 43400 Selangor, Serdang, Malaysia; ^3^Department of Pharmaceutics, Faculty of Pharmacy, Tehran University of Medical Sciences (TUMS), Tehran, Iran

## Abstract

Gastrointestinal disturbances, such as nausea and vomiting, are considered amongst the main adverse effects associated with oral anticancer drugs due to their fast release in the gastrointestinal tract (GIT). Sustained release formulations with proper release profiles can overcome some side effects of conventional formulations. The current study was designed to prepare sustained release tablets of Capecitabine, which is approved by the Food and Drug Administration (FDA) for the treatment of advanced breast cancer, using hydroxypropyl methylcellulose (HPMC), carbomer934P, sodium alginate, and sodium bicarbonate. Tablets were prepared using the wet granulation method and characterized such that floating lag time, total floating time, hardness, friability, drug content, weight uniformity, and *in vitro* drug release were investigated. The sustained release tablets showed good hardness and passed the friability test. The tablets' floating lag time was determined to be 30–200 seconds, and it floated more than 24 hours and released the drug for 24 hours. Then, the stability test was done and compared with the initial samples. In conclusion, by adjusting the right ratios of the excipients including release-retarding gel-forming polymers like HPMC K4M, Na alginate, carbomer934P, and sodium bicarbonate, sustained release Capecitabine floating tablet was formulated.

## 1. Introduction

After cardiovascular disease, cancer is the second reason for death. Prostate, lung, colon, and breast cancers are the most common forms of cancer. The present treatments for cancer include surgery, chemotherapy, hormone therapy, gene therapy, and radiation therapy. Currently, chemotherapeutic drugs are the most common type of cancer treatment. However, the administration of high doses of these drugs leads to some adverse toxic effects. As some reports indicated, many side effects, such as systemic side effects, diarrhea, and gastrointestinal problems will appear in anticancer therapy [1–5].

Many drugs, such as Anthracyclines, Taxanes (Docetaxel, Paclitaxel), Gemcitabine, Vinorelbine, Carboplatin, Trastuzumab, Lapatinib, Cyclophosphamide, Methotrexate, Adriamycin, Epirubicin, Mitoxantrone, Bevacizumab, and Capecitabine, are used in breast cancer. The use of these drugs is strongly recommended to make sure that the side effects and high dosage of these drugs are balanced [4, 6–8].

Capecitabine, 5′-deoxy-5-fluoro-N-((pentyloxy) carbonyl)-cytidine, is a fluoropyrimidine carbamate which has an antineoplastic activity. This chemical is a prodrug of 5′-deoxy-5-fluorouridine (5′-DFUR), which is enzymatically converted *in vivo* to 5-fluorouracil (5-FU). The commercial brand name is Xeloda, which has a biconvex face and a coated film (such as a light peach-colored film for 150 mg Capecitabine and peach-colored film for 500 mg Capecitabine). But both tablets have the same inactive excipients: hydroxypropyl methylcellulose, Croscarmellose sodium, magnesium stearate, microcrystalline cellulose, anhydrous lactose, and purified water. Also, the light peach- or peach-colored film contains talc, hydroxypropyl methylcellulose, titanium dioxide, and red and yellow iron oxides [9].

Important problems of Capecitabine as to the current clinical treatment are a short half-life and its rapid metabolism in the liver. Therefore, the administration of high doses of Capecitabine leads to some undesirable side effects [10]. All these problems can be resolved using sustained release. Based on previous research, since the advantages of these systems are to achieve the therapeutic concentration, the desired drug release rate prolonged drug release and reduction of the repeating dosage. Many of these problems can be resolved if sustained release is done [10, 11]. 

Sustained release (SR) tablets of anticancer drugs could not only provide an optimum plasma concentration with less frequent administration but also help decrease the side effects of conventional dosage forms, such as GIT problems [12]. This could increase the safe administration and improve patient compliance. Nowadays, some pharmaceutical products are considered as controlled-release, which can also be an effective way to deliver different types of drugs into the tissues or cells, such as diltiazem hydrochloride, chlorpheniramine maleate, ciprofloxacin, theophylline, famotidine, and captopril [5, 13–16].

There are several advantages to making sustained release antineoplastic drugs like Capecitabine. These drugs show fewer side effects, have longer half-lives, require less frequent dosages, and improve efficacy. Thus, there would be better patient compliance and less variation in plasma/blood levels [17]. The gastroretentive drug delivery system is formulated to keep the tablet in the stomach for several hours and can improve the drug's solubility and bioavailability and reduce drug waste [5, 18]. This study aimed to prepare the floating dosage form of anticancer drugs, to characterize the sustained release tablet in terms of total floating time, dissolution, friability, hardness, drug content, and weight uniformity, to compare the prepared formulation with the commercial tablet in terms of drug release, and to evaluate the stability of the formulation by accelerating and long term condition according to the International Conference on Harmonization (ICH) procedure.

## 2. Materials and Methods

### 2.1. Materials

Capecitabine was a kind gift from Osvah Pharmaceutical Company (Tehran, Iran). The 5 FU was provided from sigma Aldrich (KL, Malaysia). HPMC K4M was supplied by Sigma Chemicals. Sodium alginate and sodium bicarbonate were purchased from R&M chemicals (KL, Malaysia),carbomer934p was purchased from Noveon, polyethylene glycol 3500 from Merck, magnesium stearate from Mallinckrodt, and lactose from HMbG chemicals (United State of America). All reagents were of analytical or pharmaceutical grade.

### 2.2. Methods

#### 2.2.1. Preparation of Sustained Release Capecitabine

Sustained release tablets were formulated with different types and ratios of polymers using the wet granulation method, and then tablets were compressed directly by a single punch machine. Capecitabine was mixed with carbomer934p as a control release agent, with HPMC K4M as a binder, sodium alginate for gel forming, and sodium bicarbonate to extend floating time. All components were mixed for 10 min, and then Isopropyl alcohol was added dropwise to make a good wet mass of granules. After remixing for 5 min, the granules were passed through a 400 *μ*m mesh sieve. Wet granules were put in a 40°C oven for 40 min to become dry, and then PEG 3500 and magnesium stearate were added to the granules as lubricating agents. Eventually, 300 mg of the mixture was weighted and compressed on an 8 mm flat face by a single punch machine. In this study, 12 formulations were designed with 150 mg of Capecitabine, and the different ratios of polymers are as shown in Table 1.

#### 2.2.2. Kinetic Modeling of Release Profiles

The dissolution results of all formulations in 0.1 N HCl were specified to Higuchi, Korsmeyer-Peppas, Hixson-Crowell, Weibull, and first order and zero order kinetic models. The model with the maximum correlation coefficient was considered to be the best model [19–24].

#### 2.2.3. Determination of Floating Lag Time and Total Floating Time

The floating lag time (FLT) is the time taken for a tablet to rise on medium surface, and total floating time (TFT) is the floating duration that a tablet remained on surface. To determine the floating lag time, tablets (*n* = 4) were put on 100 mL of 0.1 N HCL in a beaker, and the time is required for a tablet to rise on surface was measured. Then, the duration of each formulation that remained on the surface was determined as total floating [15, 25]. 

#### 2.2.4. Tablet Hardness

To evaluate tablet hardness, 10 tablets of each formulation were tested for diametrical crushing strength using a hardness tester (Dr. schleuniger, 6D-Tablet Tester). 

#### 2.2.5. Tablet Friability

The friability of the SR tablets (*n* = 10) was tested by a friabilator (ERWEKA, TAR 10), at a speed of 100 rpm for 5 minutes. 

Hardness and friability values were determined and reported as mean ± SD. 

#### 2.2.6. Drug Content of the Tablets

To evaluate the drug content through a uniformity test, 10 tablets of each formulation were crushed and suspended in 0.1 N HCL to remove the Capecitabine from the tablets. After 24 hours, media were filtrated and measured by a UV spectrophotometer (Shimadzu 1601) at 214 nm [26, 27]. 

#### 2.2.7. Tablet Weight Uniformity

An electronic balance (Mettler Toledo, 3-MS-S/MS-L, Switzerland) was used to accurately weigh ten tablets which were randomly selected. The results are expressed as mean values ± SD [26, 27].

#### 2.2.8. *In Vitro* Release Study

A dissolution test was performed for 24 hours using the ERWEKA DT70 dissolution machine according to American pharmacopeia [25]. Each vessel contained 1000 mL of 0.1 N HCL; the paddle apparatus with 50 rpm speed was also used, while the temperature was kept stable at 37°C. Every two hours till 24 hours, 10 mL of media was withdrawn and measured by UV spectrophotometer at 214 nm (Shimadzu 1601). Furthermore, 10 mL of 0.1 N HCL was replaced to keep the volume stable. 

Two formulations (commercial (Xeloda) and prepared tablet) were compared in terms of drug release.

At the end, all results were analyzed using Microsoft Excel. The dissolution test was repeated 4 times for each formulation. 

#### 2.2.9. Preparation of Standard Curve

The standard curve was constructed using six different concentrations of Capecitabine, ranging from 100 to 12.5 mg. To make a standard curve, 5 mg of Capecitabine was dissolved in 50 mL of 0.1 N HCL. Then, 3 mL of each dilution was measured by UV spectrophotometer (Shimadzu 1601).

#### 2.2.10. Stability Study Test

To study the quality of the finished product under a variety of conditions (time, humidity, and temperature) and to evaluate the formulation, stability studies were prepared for 6 and 12 months according to the ICH (International Conference on Harmonization) procedures. After storage, all samples were analyzed for their physical characterizations.

Tablets (*n* = 4) were used for the stability studies according to ICH long term and accelerated procedure. All tablets were stored in standard condition in WTB binder APT line (Table 2).

All the tablets were packed in polyethylene bags. The bags were clamped using clamping tape and double-packed by putting in cardboard with a plywood lid and the lid was sealed [28–31].

#### 2.2.11. Statistical Analysis

The results were evaluated by one-way analysis of variance (ANOVA) using Duncan's multiple comparison test. Differences were considered significant at *P* value equal to or less than 0.05 [32, 33].

## 3. Results and Discussion

### 3.1. Floating Profile

The sustained release Capecitabine floating tablets were developed using release-retarding gel-forming polymers HPMC K4M, Na alginate, and carbomer934P, accompanied by sodium bicarbonate as a gas-forming agent and lactose as filler.

Table 3 shows the results of the floating and releasing times of 12 prepared formulations over 24 hours.

The investigated gastric floating systems employed sodium bicarbonate (NaHCO_3_) as a gas-forming agent, which is trapped in a hydrogel matrix (HPMC K4M and Na alginate). The *in vitro *study revealed that most formulations are able to keep the drug buoyant for more than 24 h (Table 3). This suggests that the gel layers, formed by the investigated polymers, enabled efficient entrapment of the generated CO_2_ bubbles.

The floating lag time for most formulations was below 90 seconds, regardless of the content of polymers used (Table 3), indicating significance of the polymers' concentrations (Table 1). The interaction between sodium bicarbonate (NaHCO_3_) as a gas-generating agent and the dissolution medium (0.1 mol L^−1^ HCl, pH 1.2) generated and entrapped CO_2_ inside the jellified polymeric matrices, inducing the tablet to float. A decrease in tablet-specific gravity causes the tablet to float on extended residence time in the stomach, improving absorption.

As the amount of carbomer934P increased, TFT decreased—this could be due to the high affinity of carbomer towards water, which promotes water penetration into polymeric matrices, leading to increased density. As the amount of HPMC K4M increased, the total floating time increased—this is because of the increased gel strength of the matrices, which prevents the escape of involved CO_2_ from the matrices, leading to decreased density. As the amount of SA increased, TFT decreased—this is because of the poor gelling strength of SA compared to HPMC K4M that was previously reported [34, 35].

### 3.2. Drug Release Profiles

Depending on the type and concentration of polymers, variable drug release profiles were successfully tailored.

**Figure 1:**
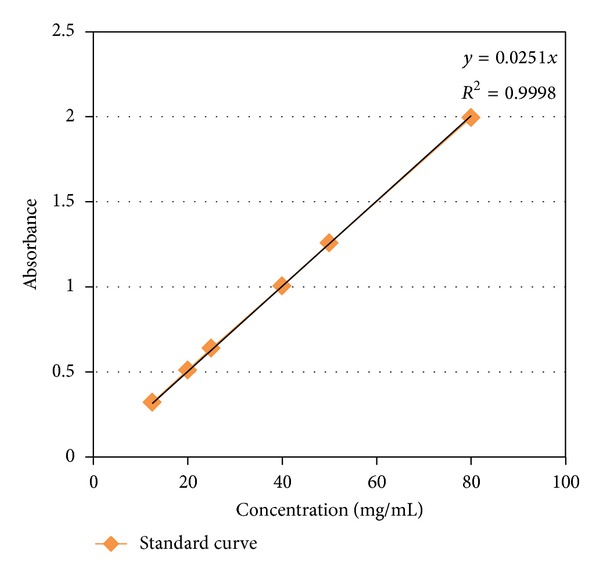
Calibration curve of Capecitabine in HCL 0.1 N.

**Figure 2:**
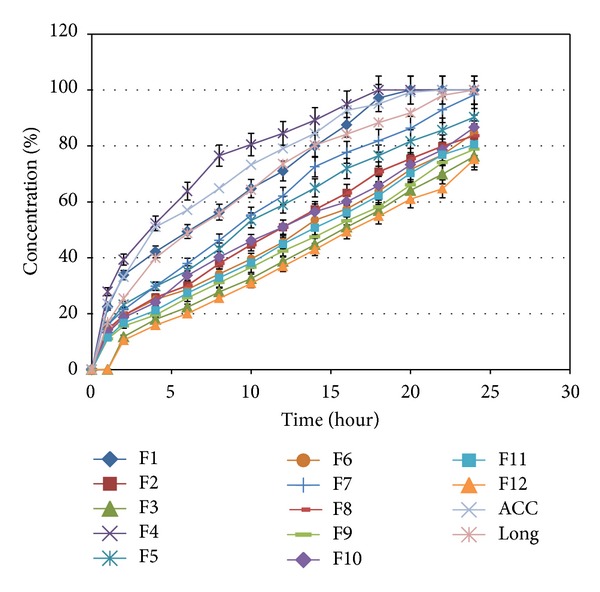
*In vitro* release profiles of various Capecitabine floating formulations in 0.1 N HCl (pH 1.2) at 37 ± 0.5°C (*n* = 4).

The dissolution profile of the best formulation (formulation F7) according to standard curve and R2 (Figure 2) was choose and is shown in Figure 1, with appropriate release rate near zero order release kinetic. Drug release involves a combination of swelling, diffusion, and erosion of matrices. This might be due to the water solubility of Capecitabine as well as different characteristics of polymers.

**Figure 3:**
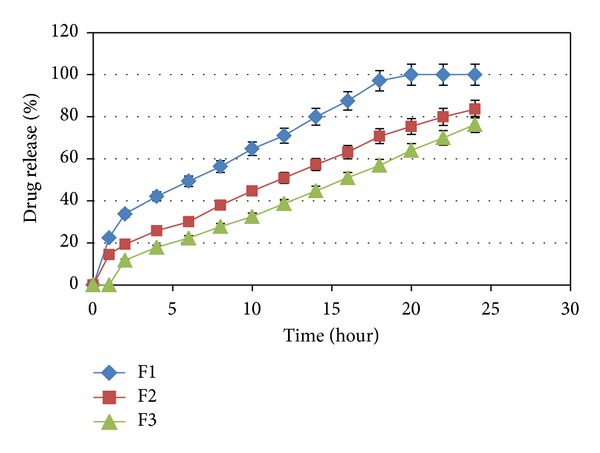
The influence of carbomer934P on the release of Capecitabine from the SR tablets in 0.1 N HCl (pH 1.2) at 37 ± 0.5°C (*n* = 4).

**Figure 4:**
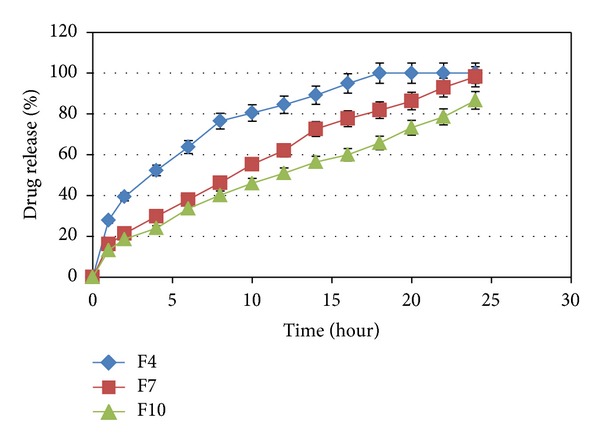
The influence of HPMC K4M in F4, F7, and F10 on the release of Capecitabine from the SR tablets in 0.1 N HCl (pH 1.2) at 37 ± 0.5°C (*n* = 4).

**Figure 5:**
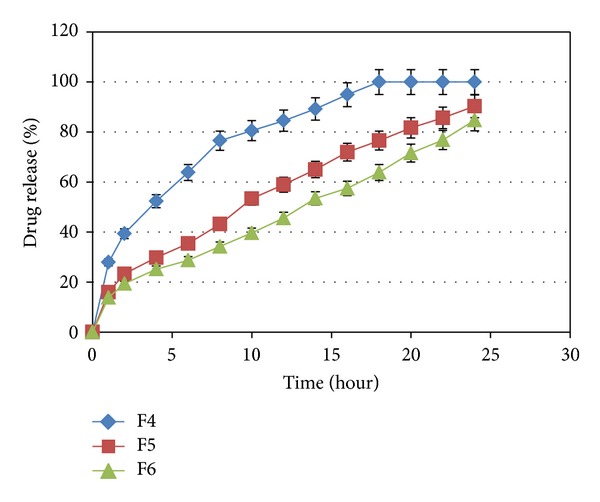
The influence of Na alginate in F4, F5, and F6 on the release of Capecitabine from the SR tablets in 0.1 N HCl (pH 1.2) at 37 ± 0.5°C (*n* = 4).

**Figure 6:**
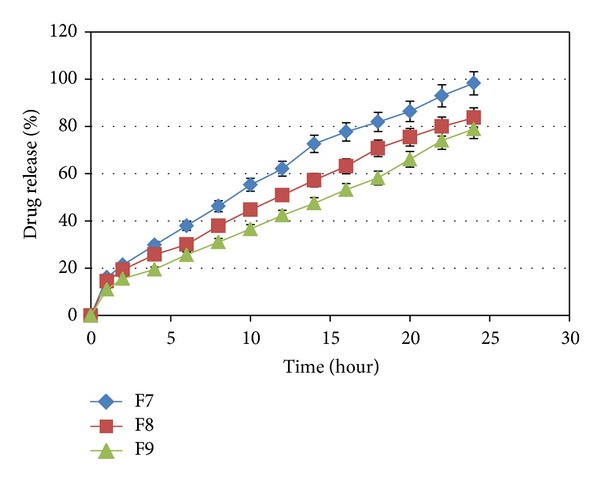
The influence of Na alginate in F7, F8, and F9 on the release of Capecitabine from the SR tablets in 0.1 N HCl (pH 1.2) at 37 ± 0.5°C (*n* = 4).

The influence of carbomer934P, HPMC K4M, and Na alginate on the release of capecitabine from the floating tablets in 0.1 N HCl (pH 1.2) at 37 ± 0.5°C was shown in Figures 3, 4, 5, and 6. It is clear that all formulations succeeded in controlling the rate of drug release. However, the drug release rate was dependent on the type and concentration of the collaborated polymers. A higher concentration of HPMC K4M would promote the formation of highly viscous gels upon contact with aqueous fluids. This would promote retardation of the drug release rates. Siepmann and Peppas [36] suggested that drug release from HPMC matrices is sequentially governed as follows: at the initial time, when the tablet contacts the media, water can penetrate into the polymeric complex, and due to water absorption, HPMC will swell and increase the dimensions of the complex. Then, drug will dissolve and diffuse out due to the concentration of the polymers.

The results of *t*_50_ (time required for 50% drug release) showed wide variations. From the results of multiple regression analysis, it was found that the dependent variable, *t*_50_, is strongly dependent on the independent variables (carbomer934P, HPMC, and Na alginate). As the amount of HPMC K4M and carbomer934P increased, *t*_50_ decreased and floating lag time increased; again, this may be due to the high affinity of HPMC and carbomer934P towards water, which promotes water penetration into polymeric matrices, leading to solubility of Capecitabine. As the amount of Na alginate increased, *t*_50_ decreased probably because of the poor water affinity of Na alginate compared to HPMC K4M and carbomer934P.

As this and previous studies [5, 37, 38] have shown that, upon contact with aqueous media, polymers would produce strong barriers that would effectively reduce the burst release. Taking into consideration the aim of the research of achieving a compromise between excellent floating behavior (short floating lag time and long total floating time) and sustained drug release characteristics, formula F7 was chosen for further studies.

**Figure 7:**
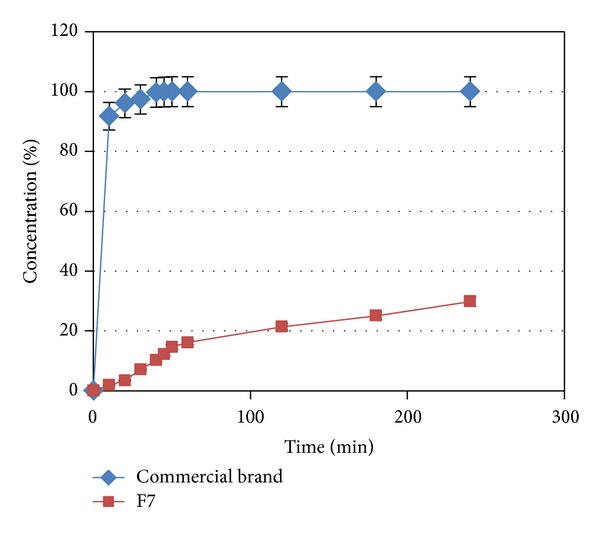
Release profiles of a commercial brand of Capecitabine and F7 (*n* = 4).

An immediate release rate was achieved following the dissolution of a commercial brand of Capecitabine 150 mg tablets in 0.1 N HCl. Indeed, 100% of the drug was released within 40 min (Figure 7). There was a significant difference between immediate release and sustained release (*P* < 0.05). The rate of immediate release significantly increased and most of the drug was released within the first 30 minutes, but in sustained release, the drug release increased gradually during 24 hours (Figure 7). 

### 3.3. Kinetic Results

To establish the mechanism of drug release, all data from the dissolution studies of floating tablets were obtained and fitted in kinetic models (Table 4) [19, 23, 24, 39]. The correlation coefficient (*R* ^2^) was used as an indicator for best fitting, in which all formulation regression values were between (*R* ^2^) = 0.998 to 0.871 zero order. By comparing the regression values of different models, the zero order model was found to be the best model for optimum formulation (F7) (Table 4). According to the results, it could be predicted that the drug release model of the prepared tablet was of the diffusion type.

### 3.4. Physical Properties

Previous studies have reported that tablet hardness not only had a slight effect on drug release profiles but was also a determining factor with regards to buoyancy of the tablets. Increasing the hardness would possibly lead to prolongation of the floating lag time by affecting the rate of the tablet penetration by the dissolution medium. Also, the percentage friability for all formulae was less than 1%, indicating good mechanical resistance. The physicochemical properties of the tablets are as summarized in Table 6.

All tablet formulae showed (Table 5) acceptable physicochemical properties and complied with the pharmacopoeia specifications [25, 26] for weight variation, drug content and friability. The weight of the tablets ranged from 298 to 301 mg.

Drug uniformity results were found to be good among different formulations, where the percentage of drug content ranged from 98.06% to 99.86%.

### 3.5. Drug Release and Physical Profile in Stability Condition

**Figure 8:**
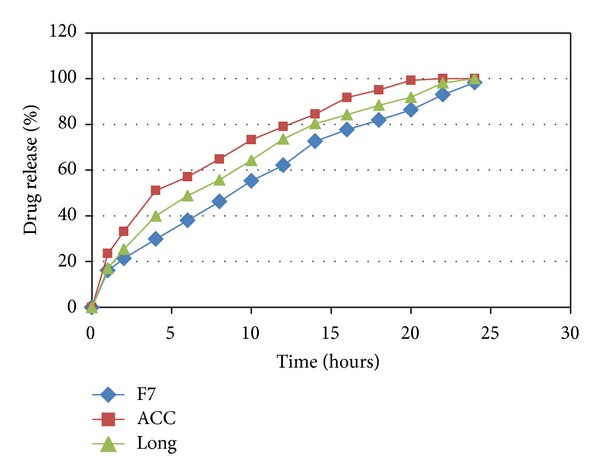
Comparison of the release profiles of F7 and stored tablets (*n* = 4).

The optimum formulation (F7) was packed according the standard procedures, and was analyzed by dissolution and physical characterization procedures after storage (Tables 2 and 6 and Figure 8).

The drug release of the stored samples was slightly affected by the different storage conditions, indicating that either heat or humidity affected the permeability of the polymeric matrix.

Accelerated test (which carried out at 40°C and 75% humidity) affected the floating ability of tablets by slight decrease in floating time.

### 3.6. Statistical Analysis Results

Before and after conducting the stability studies, statistical analyses of the results for storage months were carried out by one-way ANOVA. No significant difference (*P*  value > 0.05) was observed in the drug release.

F7 stability test after 6 months: there was no significant effect of accelerated term on F7 stability conditions at the *P* < 0.05 level for the three conditions (*F*(1,26) = 1.108138, *P* = 0.302173, and Fcrit = 4.225201).

F7 stability test after 12 months: there was no significant effect in release rate and stability of F7 after 12 months at the *P* < 0.05 level for the three conditions (*F*(1,26) = 0.285179, *P* = 0.597864, and Fcrit = 4.225201).

F7 stability test in 6 and 12 months: there was no significant effect of 6 months and 12 months on stability condition at the *P* < 0.05 level for the three conditions (*F*(1,26) = 0.260494, *P* = 0.614087, and Fcrit = 4.225201).

## 4. Conclusion

The purpose of this study was to prepare a sustained release tablet of Capecitabine with a 24-hour gradual release with concurrent floating. In doing so, various polymers, such as HPMC K4M, sodium alginate, and sodium bicarbonate, were tested. Also, characterization tests such as floating lag time, total floating time, release measurements, hardness, friability, content uniformity, and weight uniformity were performed. Comparisons of all release studies showed that the drug release depended on the ratio of two polymers—HPMC K4M, which was used as a binder; and sodium alginate, which created gel-forming capabilities in the tablet.

## Figures and Tables

**Table 1:** Different formulations of tablets with different concentrations (%).

	HPMC	S.A	Car	S.B	Lac	PEG	Mg.St	Cap	Total
F1	20	20	1.6	13.3	35	6.6	3.3	150	300
F2	20	20	3.3	13.3	33.3	6.6	3.3	150	300
F3	20	20	4.6	13.3	32	6.6	3.3	150	300
F4	16.6	16.6	3.3	13.3	40	6.6	3.3	150	300
F5	16.6	20	3.3	13.3	36.6	6.6	3.3	150	300
F6	16.6	23.3	3.3	13.3	33.3	6.6	3.3	150	300
F7	20	16.6	3.3	13.3	36.6	6.6	3.3	150	300
F8	20	20	3.3	13.3	33.3	6.6	3.3	150	300
F9	20	23.3	3.3	13.3	30	6.6	3.3	150	300
F10	23.3	16.6	3.3	13.3	33.3	6.6	3.3	150	300
F11	23.3	20	3.3	13.3	30	6.6	3.3	150	300
F12	23.3	23.3	3.3	13.3	26.6	6.6	3.3	150	300

**Table 2:** Storage conditions for tablet stability test.

Type of study	Condition	Time
Accelerated	40°C ± 2°C, 75% RH ± 5% RH	6 months
Long term	25°C±2°C, 60% RH ± 5% RH	12 months

**Table 3:** Drug release and floating profiles of twelve formulations.

Formulation	Release %	Floating lag time (s)	Total floating time (h)
F1	100	30	20
F2	83.665	70	24
F3	76.3	81	24
F4	100	35	20
F5	90.305	45	24
F6	84.711	60	24
F7	98.286	60	24
F8	83.665	70	24
F9	78.82	85	24
F10	86.666	60	24
F11	80.54	80	24
F12	75.226	200	0.5

s: second; hr: hour.

**Table 4:** Mathematical release modeling of sustained release capecitabine floating tablets.

Formulations code	Zero order *R* ^2^	First order *R* ^2^	Higuchi *R* ^2^	Hixson-Crowell *R* ^2^	Korsmeyer-Peppas
F1	0.964	0.818	0.983	0.908	0.482
F2	0.996	0.972	0.977	0.990	0.576
F3	0.992	0.959	0.970	0.980	1.606
F4	0.871	0.974	0.962	0.940	0.413
F5	0.989	0.968	0.988	0.993	0.558
F6	0.996	0.926	0.954	0.963	0.564
F7	0.989	0.851	0.988	0.960	0.596
F8	0.996	0.972	0.977	0.990	0.575
F9	0.997	0.943	0.955	0.969	0.625
F10	0.993	0.936	0.980	0.973	0.590
F11	0.998	0.951	0.962	0.976	0.621
F12	0.992	0.954	0.967	0.976	1.644
Acc	0.922	0.857	0.986	0.955	0.464
Long	0.955	0.894	0.997	0.918	0.464

**Table 5:** Comparison of physical properties of all formulations.

	Hardness (N)	Friability (%)	Drug content (%)	Weight uniformity (mg)
F1	57	0.35	99.33 ± 0.81	299 ± 0.89
F2	76	0.26	98.61 ± 1.13	300 ± 0.75
F3	81	0.22	99.45 ± 0.19	301 ± 0.82
F4	55	0.35	99.83 ± 0.88	298 ± 1.04
F5	62	0.29	98.17 ± 1.05	299 ± 0.79
F6	68	0.25	99.05 ± 0.71	299 ± 0.9
F7	69	0.31	99.79 ± 0.48	299 ± 0.18
F8	76	0.26	98.61 ± 1.13	298 ± 0.62
F9	80	0.21	99.86 ± 0.36	300 ± 0.25
F10	95	0.19	99.25 ± 0.51	298 ± 0.72
F11	104	0.11	98.48 ± 0.19	300 ± 0.43
F12	112	0.103	99.01 ± 0.47	299 ± 1.09

**Table 6:** Drug release and physical properties of stored tablet.

Formulation	Release %	Floating lag time	Total floating time	Hardness (N)	Friability (%)	Drug content (%)	Weight uniformity (mg)
ACC	99.216	80 s	20 h	57	0.30	98.06 ± 0.61	298 ± 0.41
Long	100	65 s	23 h	65	0.33	99.37 ± 0.82	299 ± 0.16

## Abbreviations

*μ*g: Microgram
ACC: Accelerated time
API: Active pharmaceutical ingredient
BP: British pharmacopeia
Cap: Capecitabine
Car: Carbomer934p
FDA: Food and Drug Administration
HPMC: Hydroxypropyl methylcellulose
Lac: Lactose
Long: Long term
mg: Milligram
Mg.St: Magnesium stearate
Min: Minute
mm: Millimeter
°C: Degree Celsius
PEG: PolyEthylene glycol
RH: Relative humidity
S.A: Sodium alginate
S.B: Sodium bicarbonate
USP: United States of pharmacopeia
UV: Ultraviolet.
